# Immune evasion by *Staphylococcus haemolyticus*: Attenuated NET-associated responses and resistance to NET-mediated killing

**DOI:** 10.3934/microbiol.2026021

**Published:** 2026-07-27

**Authors:** Mohamed Ahmed, Marwa M. Khalifa

**Affiliations:** 1 College of Medicine, Dhofar University, Salalah 211, Sultanate of Oman; 2 Department of Microbiology and Immunology, Faculty of Pharmacy, Assiut University, Assiut, Egypt

**Keywords:** *Staphylococcus haemolyticus*, coagulase-negative staphylococci, neutrophil extracellular traps, NET-associated response, immune evasion, polymorphonuclear neutrophils, healthcare-associated infections

## Abstract

Neutrophil extracellular traps (NETs) contribute to innate immune defense by trapping and exposing microorganisms to DNA-associated antimicrobial proteins. However, some pathogens can tolerate NET-associated antimicrobial activity and persist within the host. *Staphylococcus haemolyticus* is an emerging multidrug-resistant coagulase-negative staphylococcal pathogen associated with healthcare-related infections, but its interaction with neutrophil-mediated defenses remains poorly understood. This study investigated whether a multidrug-resistant clinical *S. haemolyticus* isolate modulates neutrophil antimicrobial activity and NET-associated responses, using a clinical *Staphylococcus aureus* isolate as a comparator. Extracellular DNA release and NET-associated structures were assessed by fluorescence microscopy, scanning electron microscopy, fluorometric DNA quantification, microscopic enumeration, and MPO-DNA complex ELISA. The tested *S. haemolyticus* isolate induced extracellular DNA release and MPO-DNA complex formation; however, these responses were lower than those induced by PMA and were attenuated compared with the *S. aureus* comparator. *S. haemolyticus* also showed reduced susceptibility to neutrophil-mediated killing and greater resistance to NET-mediated killing than *S. aureus*. NE and MPO mRNA expression was not significantly altered following bacterial exposure. Collectively, these findings provide initial evidence that a clinical *S. haemolyticus* isolate can induce attenuated NET-associated responses while resisting NET-mediated antimicrobial activity, highlighting a potential immune-evasion mechanism in this emerging opportunistic pathogen.

## 1. Introduction

*Staphylococcus haemolyticus* belongs to the group of coagulase-negative staphylococci (CoNS), which includes most species within the genus *Staphylococcus* [1],[2]. CoNS are common inhabitants of human skin and mucosal surfaces and form part of the normal microbiota. However, some species, including *S. epidermidis*, *S. haemolyticus*, and *S. saprophyticus*, have emerged as important opportunistic pathogens, particularly in hospitalized patients, immunocompromised individuals, and patients exposed to invasive medical procedures or indwelling devices. CoNS-associated infections range from superficial skin and soft tissue infections to serious systemic diseases, including bacteremia and endocarditis [3]–[5].

*S. haemolyticus* remains an underrecognized clinical threat, as the skin and mucosal surfaces can serve as entry sites for this opportunistic pathogen. It has been implicated in a wide range of infections, particularly in immunocompromised patients and individuals with implanted medical devices. Reported infections include meningitis, bacteremia, septicemia, prosthetic joint infections, otitis, peritonitis, and neonatal sepsis [6]. Multidrug-resistant *S. haemolyticus* strains are increasingly associated with bloodstream infections and endocarditis [7]–[9], as well as sepsis in neonates [10]. *S. haemolyticus* can also cause skin and soft tissue infections [11],[12], and less commonly, urinary tract infections [13],[14], meningitis [15], and ocular and laryngological infections [16],[17].

Neutrophils, also known as polymorphonuclear leukocytes (PMNs), are essential cellular components of the innate immune response and play a central role in host defense against bacterial infections [18]. Neutrophils eliminate invading microorganisms through several antimicrobial mechanisms, including the release of antimicrobial compounds, production of reactive oxygen and nitrogen species, phagocytosis, and formation of neutrophil extracellular traps (NETs) [19]. NETs are delicate extracellular web-like structures composed of DNA fibers decorated with proteases, antimicrobial peptides, histones, and cytoskeletal proteins, and contribute to host protection against bacterial, fungal, and viral infections [20]. NET formation is characterized by chromatin decondensation and the release of DNA into the extracellular environment, where it forms web-like structures capable of trapping microorganisms. In general, NETs contribute to antimicrobial defense by immobilizing pathogens and exposing them to NET-associated antimicrobial proteins, including neutrophil elastase (NE) and myeloperoxidase (MPO) [21],[22].

The widespread distribution of *S. haemolyticus* across diverse environments highlights its relevance within the One Health framework and emphasizes the need for further research and targeted strategies to control this adaptable microorganism. Owing to its emergence as a nosocomial pathogen and its alarming antimicrobial resistance profile, *S. haemolyticus* has attracted increasing research attention. In this context, the present study aimed to investigate neutrophil responses to a clinical *S. haemolyticus* isolate, with particular focus on extracellular DNA release, NET-associated responses, and resistance to NET-mediated killing.

## 2. Materials and methods

### 2.1. Ethics statement

The study protocol was approved by the Ethics Committee of the Faculty of Medicine, Assiut University, Assiut, Egypt (approval no. 17-300-379). The study was conducted in accordance with the Declaration of Helsinki. Written informed consent was obtained from all participants.

### 2.2. Bacterial isolates

The *S. haemolyticus* isolate used in this study was selected from a previously characterized diabetic foot ulcer clinical strain collection. Species-level identification had been performed using the VITEK® 2 automated microbiology system (bioMérieux, Marcy-l'Étoile, France) and further confirmed by multiplex PCR, as previously described [6]. A local clinical *S. aureus* isolate recovered from the same diabetic foot ulcer patient population was used as a clinically relevant comparator.

### 2.3. Neutrophil isolation protocol

Neutrophils were isolated from freshly collected peripheral blood of healthy volunteers using a previously published protocol based on gelatin sedimentation, Ficoll density-gradient separation, and brief hypotonic erythrocyte lysis, with minor modifications [23]. Briefly, erythrocytes were sedimented using sedimentation fluid containing 3% gelatin, 0.9% NaCl, and 0.1% dextrose. The leukocyte-rich plasma was then layered onto Ficoll-Paque PLUS (Cytiva, Sweden, catalog number: 17-1440-02) and centrifuged at 400× g for 20 min at 22 °C.

After removal of the supernatant, the polymorphonuclear cell/red blood cell pellet was subjected to a brief hypotonic lysis step to eliminate residual erythrocytes. The pellet was resuspended in 5 mL of distilled water and 1 mL of PBS for 45 s with gentle inversion. Hypotonic lysis was immediately stopped by adding 2 mL of 3% NaCl to restore tonicity. The suspension was then centrifuged at 1500 rpm for 5 min at 22 °C. The supernatant was discarded, and the neutrophil pellet was gently resuspended in RPMI 1640 medium. Cell viability was assessed by trypan blue exclusion, and cell morphology was examined using 0.1% methylene blue staining before use in functional assays [23].

### 2.4. Neutrophil killing assays

Serum-opsonized bacteria (1 × 10^6^ CFU) were incubated with isolated neutrophils (1 × 10^6^ cells) at a 1:1 ratio in microcentrifuge tubes at 37 °C with gentle rotation for 60 min. To assess the contribution of NET-mediated killing, parallel samples were treated with DNase I (SERVA Electrophoresis GmbH, Heidelberg, Germany; catalog number: 001853501) for 10 min before incubation to degrade extracellular DNA and disrupt NET-associated antibacterial activity. After incubation, neutrophils were lysed with 0.1% Triton X-100 (Acros Organics, Thermo Fisher Scientific, Geel, Belgium) for 15 min on ice, and viable bacterial counts were determined by plating serial dilutions on LB agar. Bacterial growth in neutrophil-free RPMI 1640 medium was included as a control. Bacterial survival was calculated as the percentage of CFU recovered after neutrophil exposure relative to the neutrophil-free control [24].

### 2.5. Neutrophil phagocytosis assays

#### 2.5.1. Fluorescence labeling of bacteria

Bacterial isolates were cultured overnight at 37 °C, and bacterial concentrations were determined by quantitative plate counting. Bacteria were heat-inactivated at 70 °C for 1 h, washed with PBS, and labeled with fluorescein isothiocyanate (FITC; Acros Organics, Thermo Fisher Scientific, Geel, Belgium). Briefly, bacteria were incubated with 0.1 mg/mL FITC in 0.10 M NaHCO_3_, pH 9.0, for 60 min at 25 °C. FITC-labeled bacteria were then washed three times with PBS and centrifuged to remove unbound fluorochrome. The labeled bacteria were resuspended in PBS at a final concentration of 2 × 10^8^ cells/mL, aliquoted, and stored at −70 °C until use [25].

#### 2.5.2. Phagocytosis assay

A prewarmed reaction mixture containing 100 µL of pooled normal human serum for opsonization, 100 µL of neutrophil suspension (1 × 10^6^ cells), and 600 µL of PBS was combined with 200 µL of FITC-labeled bacterial suspension (4 × 10^7^ cells). The final serum concentration was 10% (v/v). Tubes were incubated in a shaking water bath at 37 °C, except for unincubated control tubes, which were used as the 0-min control. After incubation for 10, 30, or 60 min, tubes were immediately placed in a cold bath to stop phagocytosis. The percentage of neutrophils associated with FITC-labeled bacteria was assessed by flow cytometry using a FACS Calibur (BD Biosciences, San Jose, CA, USA). Neutrophils were gated based on forward scatter (FSC) and side scatter (SSC) characteristics. A total of 50,000 neutrophil events were acquired and analyzed for each sample [26]. Before flow cytometric acquisition, trypan blue was added to quench extracellular fluorescence from surface-bound FITC-labeled bacteria, allowing assessment of internalized bacteria.

### 2.6. Visualization of NET-associated structures by scanning electron microscopy

Neutrophils (1 × 10^6^ cells/well) were seeded onto sterile coverslips placed in 12-well flat-bottom plates and allowed to adhere. Cells were then treated with PBS as a negative control or phorbol 12-myristate 13-acetate (PMA; Abcam, Cambridge, UK) at 100 ng/mL as a positive control or challenged with 200 µL of bacterial suspension (4 × 10^7^ CFU/mL). Plates were incubated for 4 h at 37 °C in a humidified atmosphere containing 5% CO_2_.

After incubation, neutrophils were fixed with 3% glutaraldehyde and post-fixed with 1% osmium tetroxide. Samples were then dehydrated through a graded ethanol series, subjected to critical-point drying, coated with gold, and examined using a JEOL JSM-5400LV scanning electron microscope (JEOL Ltd., Tokyo, Japan) [26].

### 2.7. Visualization and microscopic quantification of extracellular DNA release by fluorescence microscopy

Neutrophils (1 × 10^6^ cells/mL) were seeded onto sterile coverslips in 24-well plates and incubated with bacterial suspensions (4 × 10^7^ CFU/mL) in RPMI 1640 medium for 4 h at 37 °C in a humidified atmosphere containing 5% CO_2_. PBS-treated neutrophils were used as a negative control, while neutrophils stimulated with 100 nM phorbol 12-myristate 13-acetate (PMA; Abcam, Cambridge, UK) were used as a positive control [27]. After incubation, cells were fixed with 4% paraformaldehyde overnight at 4 °C and then washed three times with PBS [28]. Extracellular DNA was stained using SYTOX Green nucleic acid stain (Invitrogen, Thermo Fisher Scientific, Waltham, MA, USA) and visualized using a fluorescence microscope.

For microscopic quantification, extracellular DNA release was assessed in five randomly selected microscopic fields per coverslip. Neutrophils were considered positive when decondensed or extracellular DNA structures extending beyond the cell body were observed by SYTOX Green staining. Results were expressed as the percentage of extracellular DNA-releasing neutrophils relative to the total number of neutrophils counted in the analyzed fields.

### 2.8. Quantification of extracellular DNA release by fluorometry

Neutrophils (1 × 10^6^ cells/well) were co-cultured with bacteria (4 × 10^7^ CFU/well) in RPMI 1640 medium for 4 h at 37 °C in a humidified atmosphere containing 5% CO_2_. In parallel experiments, neutrophils were stimulated with 100 nM phorbol 12-myristate 13-acetate (PMA; Abcam, Cambridge, UK) as a positive control, while PBS-treated unstimulated neutrophils were used as a negative control.

After incubation, micrococcal nuclease (MNase; Thermo Scientific, Thermo Fisher Scientific, Waltham, MA, USA) was added to each well at a final concentration of 1 U/mL and incubated for 20 min at 37 °C to release extracellular DNA into the supernatant. The reaction was stopped by adding EDTA to a final concentration of 5 mM. Supernatants were then collected, and SYTOX Green nucleic acid stain (Invitrogen, Thermo Fisher Scientific, Waltham, MA, USA) was added to each sample at a final concentration of 1 µM.

Fluorescence intensity was measured immediately using a Jasco FP-6300 WRE spectrofluorometer (Jasco Corporation, Tokyo, Japan) at an excitation wavelength of 504 nm and an emission wavelength of 523 nm. Background fluorescence from bacteria-only wells containing bacteria in PBS without neutrophils was subtracted from the corresponding samples. Extracellular DNA release was expressed as fluorescence intensity after background subtraction [29].

### 2.9. NET-mediated killing assay

To assess NET-mediated non-phagocytic killing, isolated neutrophils (1 × 10^6^ cells/well) were suspended in 500 µL of RPMI 1640 medium and seeded into 24-well plates. NET formation was induced by stimulation with 100 nM PMA for 3 h at 37 °C in a humidified atmosphere containing 5% CO_2_.

After incubation, the supernatant was carefully removed and replaced with 500 µL of prewarmed RPMI 1640 medium, with or without 1 U/mL MNase. Plates were incubated at 37 °C for 15 min. Bacteria (4 × 10^7^ CFU/well) were then added to the appropriate wells, and plates were centrifuged at 700× g for 10 min to enhance contact between bacteria and NET structures. Plates were subsequently incubated for 1 h at 37 °C in 5% CO_2_.

Following incubation, MNase was added to each well, and plates were incubated for an additional 15 min at 37 °C to release NET-associated bacteria. The contents of each well were transferred to 1.5 mL microcentrifuge tubes and serially diluted in broth. Bacterial viability was determined by plating 10 µL of each suspension onto agar plates, followed by incubation for 24 h at 37 °C. NET-mediated non-phagocytic killing was calculated using the following Eq 1 [27]:

NET-mediated killing (%)=[(CFU with MNase−CFU without MNase)/CFU with MNase]×100. (1)

### 2.10. Quantification of MPO-DNA complexes by ELISA

MPO-DNA complexes were quantified using a modified capture ELISA [30],[31]. Neutrophils (1 × 10^6^ cells/well) were incubated for 4 h at 37 °C with PBS, PMA, *S. aureus*, or *S. haemolyticus*. MNase (1 U/mL) was then added for 20 min to release extracellular DNA-protein complexes, and the reaction was stopped with 5 mM EDTA. Supernatants were collected and centrifuged at 300× g for 5 min.

Then, 96-well plates were coated overnight at 4 °C with mouse anti-human MPO antibody (clone 4A4; Bio-Rad), washed, and blocked with 1% BSA for 1 h. Samples were added and incubated for 2 h at room temperature. Bound DNA was detected using the peroxidase-conjugated anti-DNA antibody supplied with the Cell Death Detection ELISA PLUS kit (Sigma-Aldrich, Germany) according to the manufacturer's instructions. After washing, TMB substrate was added, the reaction was stopped with 2 N H_2_SO_4_, and absorbance was measured at 450 nm. Results were normalized to PBS-treated neutrophils and expressed as fold change relative to PBS. PMA and PBS served as positive and negative controls, respectively. Measurements were performed in five replicates per condition.

### 2.11. Quantification of NE and MPO mRNA expression following NET induction

The mRNA expression of NET-associated genes, including neutrophil elastase (NE) and myeloperoxidase (MPO), was quantified by quantitative real-time PCR in neutrophils treated with bacteria or PMA. Total RNA was extracted from treated neutrophils (1 × 10^6^ cells/mL) using an RNA isolation kit (Applied Biotechnology, Egypt) according to the manufacturer's instructions. cDNA was synthesized using the ABT 2X RT Mix cDNA Synthesis kit, following the manufacturer's protocol.

Quantitative real-time PCR was performed in duplicate in a final reaction volume of 25 µL containing 100 ng of cDNA, 500 nM of each primer, and 1× SYBR Green PCR master mix. The cycling conditions were as follows: Initial denaturation at 95 °C for 5 min, followed by 38 cycles of 95 °C for 20 s, 55 °C for 30 s, and 72 °C for 30 s. The primer sequences were as follows: NE forward, 5′-GTGGCGAATGTAAACGTC-3′; NE reverse, 5′-CCGTTGAGCTGGAGAATC-3′; MPO forward, 5′-TGCTTCCTGGCAGGGGA-3′; MPO reverse, 5′-CCACCTAGGGTTCAGGCTCT-3′; GAPDH forward, 5′-AGGGCCCTGACAACTCTTTT-3′; and GAPDH reverse, 5′-TTACTCCTTGGAGGCCATGT-3′ [32]. Relative gene expression was normalized to GAPDH [32].

### 2.12. Statistical analysis

Statistical analyses were performed using GraphPad Prism version 8.0 (GraphPad Software, San Diego, CA, USA). Data were expressed as mean ± standard deviation (SD) or mean ± standard error of the mean (SEM), as indicated in the corresponding figure legends. Comparisons between two independent groups were performed using either the Mann–Whitney U test or an unpaired two-tailed Student's *t*-test, as specified in the figure legends. For comparisons among multiple groups, one-way analysis of variance (ANOVA) was used, followed by the appropriate post hoc multiple-comparison test when applicable. Differences were considered statistically significant at *p* < 0.05.

## 3. Results

### 3.1. *S. haemolyticus* shows increased survival following neutrophil exposure

To evaluate neutrophil-mediated bacterial killing, isolated human neutrophils were co-cultured with serum-opsonized bacteria at 37 °C with gentle rotation for 1 h. Bacterial survival was calculated as the percentage of CFU recovered after neutrophil exposure relative to the neutrophil-free growth control. As shown in Figure 1, *S. haemolyticus* showed significantly higher survival after exposure to neutrophils than the clinical *S. aureus* comparator isolate (*p* = 0.008). The survival percentage of *S. haemolyticus* was 64.0% ± 13.4%, whereas only 27.6% ± 2.3% of *S. aureus* survived under the same experimental conditions.

**Figure 1.**
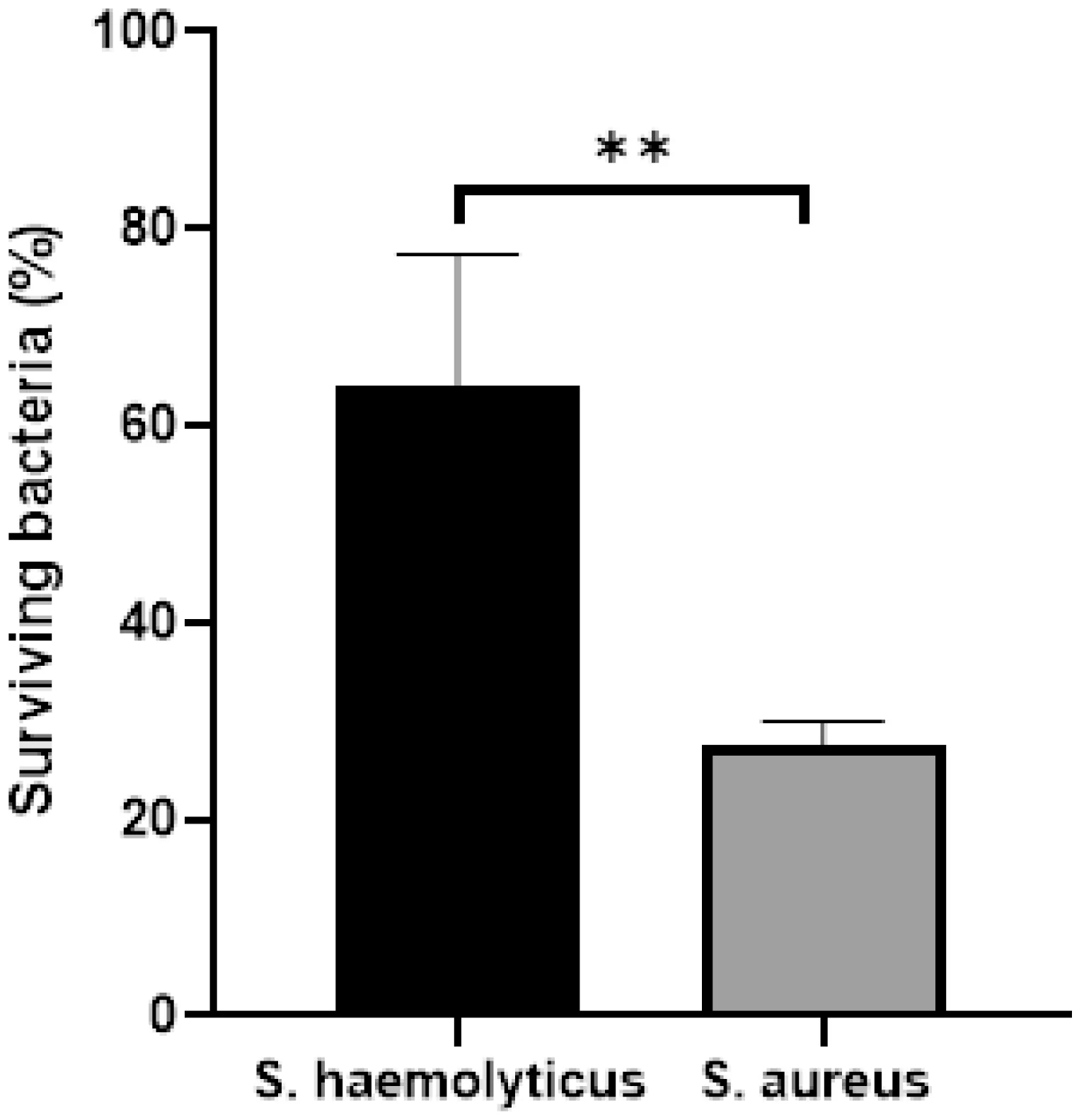
Survival of *S. haemolyticus* and *S. aureus* following neutrophil exposure. Serum-opsonized bacteria were incubated with isolated human neutrophils for 1 h at 37 °C. Bacterial survival was calculated as the percentage of CFU recovered after neutrophil exposure relative to the neutrophil-free growth control. Data are presented as mean ± SD, n = 5. **p < 0.01, Mann–Whitney U test.

### 3.2. *S. haemolyticus* shows delayed early phagocytosis by neutrophils

Phagocytosis is a major neutrophil antimicrobial mechanism that contributes to bacterial recognition, uptake, and intracellular killing. To assess whether the reduced susceptibility of *S. haemolyticus* to neutrophil-mediated killing was associated with altered phagocytic uptake, isolated human neutrophils were incubated with FITC-labeled heat-inactivated *S. haemolyticus* in the presence of normal human serum and analyzed by flow cytometry at 10, 30, and 60 min. A clinical *S. aureus* isolate was included as a comparator.

Representative flow cytometry plots at 10 min are shown in Figure 2a, and the quantitative time-course analysis is shown in Figure 2b. At the early 10-min time point, phagocytosis of *S. haemolyticus* was significantly lower than that of *S. aureus* (13.3% ± 4.6% vs. 29.2% ± 3.5%, respectively; *p* = 0.0089). At 30 min, phagocytosis of *S. haemolyticus* remained lower than that of *S. aureus*, although this difference did not reach statistical significance (28.5% ± 3.9% vs. 37.3% ± 5.2%, respectively; *p* = 0.0790). By 60 min, phagocytosis was comparable between *S. haemolyticus* and *S. aureus* (39.9% ± 5.7% vs. 43.6% ± 6.8%, respectively; *p* = 0.51).

**Figure 2.**
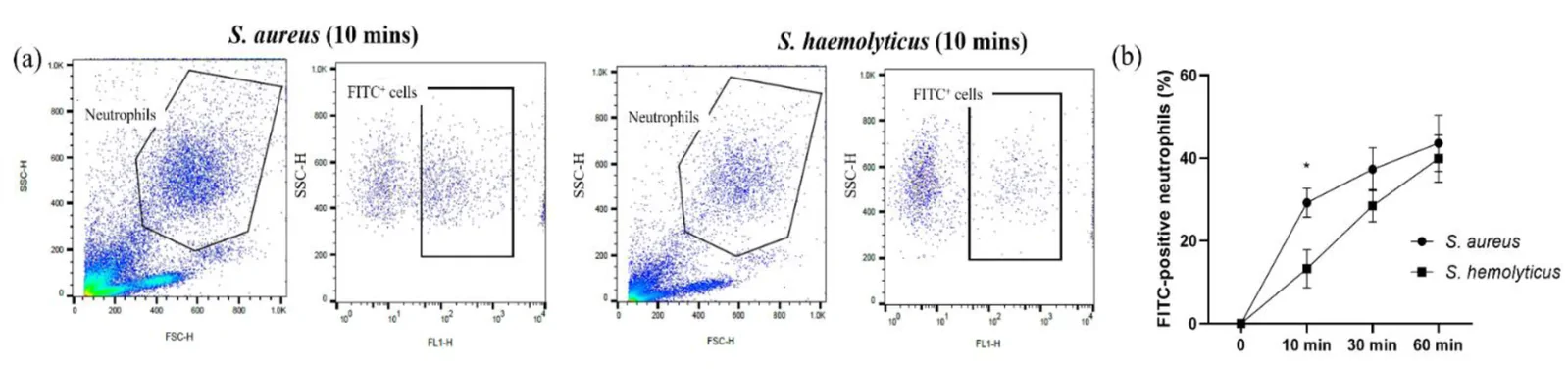
Flow cytometric analysis of neutrophil phagocytosis of FITC-labeled *S. haemolyticus*. Human neutrophils were incubated with FITC-labeled heat-inactivated *S. haemolyticus* in the presence of normal human serum and analyzed by flow cytometry, with a clinical *S. aureus* isolate included as a comparator. Trypan blue was used to quench extracellular fluorescence from surface-bound bacteria before acquisition. (a) Representative flow cytometry dot plots after 10 min of incubation showing neutrophil gating based on FSC/SSC characteristics, followed by quantification of FITC-positive neutrophils. (b) Quantitative analysis of FITC-positive neutrophils after 0, 10, 30, and 60 min of incubation. Data are presented as mean ± SD. * indicates a *p*-value less than 0.05 compared with *S. aureus* at the same time point.

### 3.3. Visualization of NET-associated structures by SEM

Scanning electron microscopy was used to examine morphological changes in neutrophils following stimulation with PMA or exposure to bacteria. As shown in Figure 3a, untreated neutrophils maintained a rounded morphology and showed no visible extracellular fibrillary structures. In contrast, PMA-stimulated neutrophils showed clear morphological activation, with cell flattening and the release of web-like extracellular fibrillary structures (Figure 3b).

Exposure to *S. aureus* also induced visible extracellular fibrillary structures associated with neutrophils and bacterial cells (Figure 3c). Similarly, neutrophils exposed to *S. haemolyticus* displayed morphological activation and extracellular web-like material surrounding bacterial clusters (Figure 3d). These SEM observations indicate that *S. haemolyticus* was able to trigger neutrophil morphological changes and NET-like extracellular structures, supporting its interaction with NET-associated neutrophil responses.

**Figure 3.**
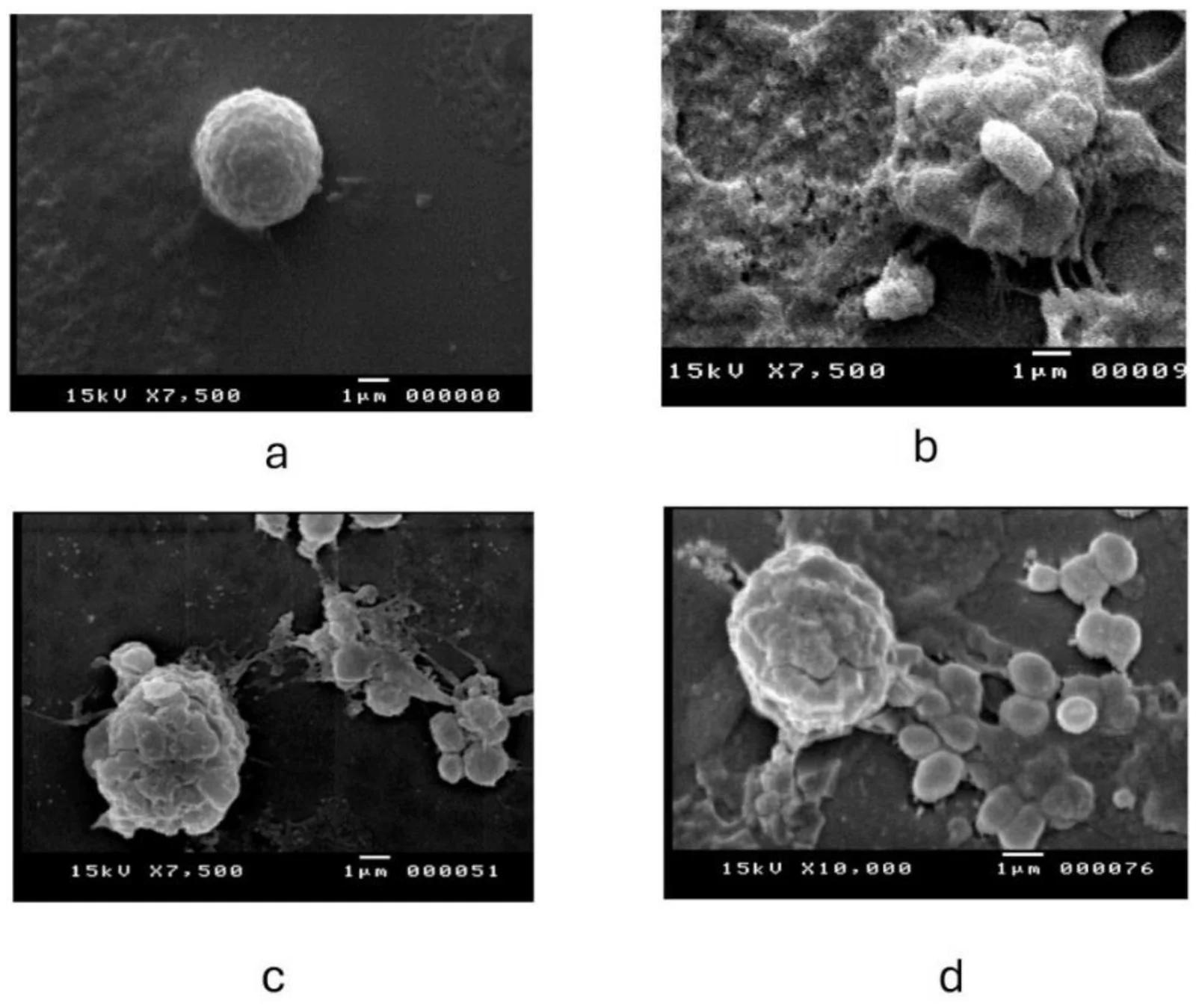
Scanning electron microscopic visualization of NET-associated structures. Neutrophils were incubated for 4 h with PBS, PMA, *S. aureus*, or *S. haemolyticus*. (a) PBS-treated neutrophil showing rounded morphology without visible extracellular fibrillary structures. (b) PMA-stimulated neutrophils showing cell flattening and web-like extracellular fibrillary structures. (c) Neutrophils exposed to *S. aureus* showing extracellular fibrillary material associated with bacteria. (d) Neutrophils exposed to *S. haemolyticus* showing extracellular web-like material surrounding bacterial cells. Representative SEM images are shown.

### 3.4. Visualization and microscopic quantification of NET-associated extracellular DNA release

NET-associated extracellular DNA release was visualized by fluorescence microscopy using SYTOX Green staining. As shown in Figure 4a, PBS-treated neutrophils showed minimal extracellular DNA release. In contrast, PMA stimulation induced marked extracellular DNA structures consistent with robust NET formation (Figure 4b). Exposure to *S. aureus* and *S. haemolyticus* also induced NET-associated extracellular DNA release compared with the PBS negative control, although the response was less extensive than that observed after PMA stimulation (Figure 4c,d).

Microscopic quantification of five randomly selected fields per condition supported the qualitative fluorescence microscopy observations (Figure 4e). PMA induced the highest percentage of neutrophils showing NET-associated extracellular DNA release (71.52% ± 6.90%). The tested *S. aureus* and *S. haemolyticus* isolates induced intermediate levels of NET-associated extracellular DNA release (43.86% ± 5.37% and 35.38% ± 5%, respectively), compared with minimal release in PBS-treated neutrophils (3.80% ± 0.81%). These findings suggest that although the tested clinical *S. haemolyticus* isolate was able to trigger NET-associated extracellular DNA release, it induced a comparatively attenuated response.

**Figure 4.**
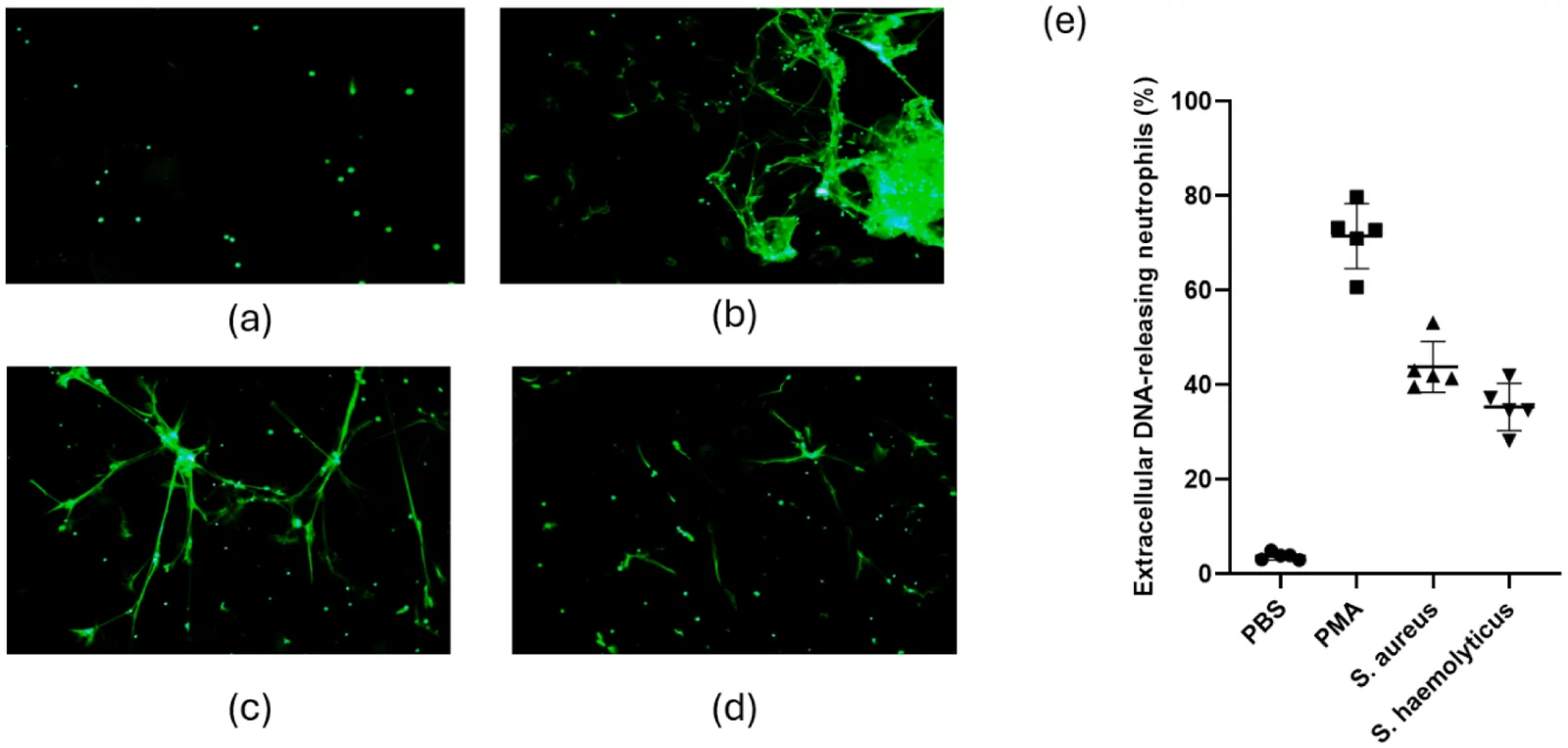
Fluorescence microscopy visualization and quantification of NET-associated extracellular DNA release by neutrophils. Neutrophils were incubated for 4 h with (a) PBS, (b) PMA, (c) *S. aureus*, or (d) *S. haemolyticus*. NET-associated extracellular DNA was visualized using SYTOX Green staining. (e) Quantification of neutrophils showing NET-associated extracellular DNA release from five randomly selected microscopic fields per condition. Data are shown as individual field values with mean ± SD.

### 3.5. Quantification of NET-associated extracellular DNA release

To further evaluate NET-associated responses, extracellular DNA release was quantified by measuring SYTOX Green fluorescence in neutrophil culture supernatants. Untreated neutrophils were used as a negative control, while PMA-stimulated neutrophils were used as a positive control for NET induction. Bacteria-only wells were included to correct for background fluorescence and to avoid overestimation due to bacterial DNA.

As shown in Figure 5, PMA stimulation induced the highest NET-associated extracellular DNA signal. Neutrophils exposed to *S. aureus* or *S. haemolyticus* also showed increased extracellular DNA release compared with PBS-treated neutrophils; however, the fluorescence intensity was significantly lower than that observed after PMA stimulation. The NET-associated extracellular DNA signal was significantly reduced in the *S. aureus* group compared with PMA (*p* = 0.0022) and was also significantly reduced in the *S. haemolyticus* group compared with PMA (*p* = 0.0411). These findings suggest that the tested clinical *S. haemolyticus* isolate can trigger NET-associated extracellular DNA release, but the magnitude of this response is attenuated compared with PMA-induced NET formation.

**Figure 5.**
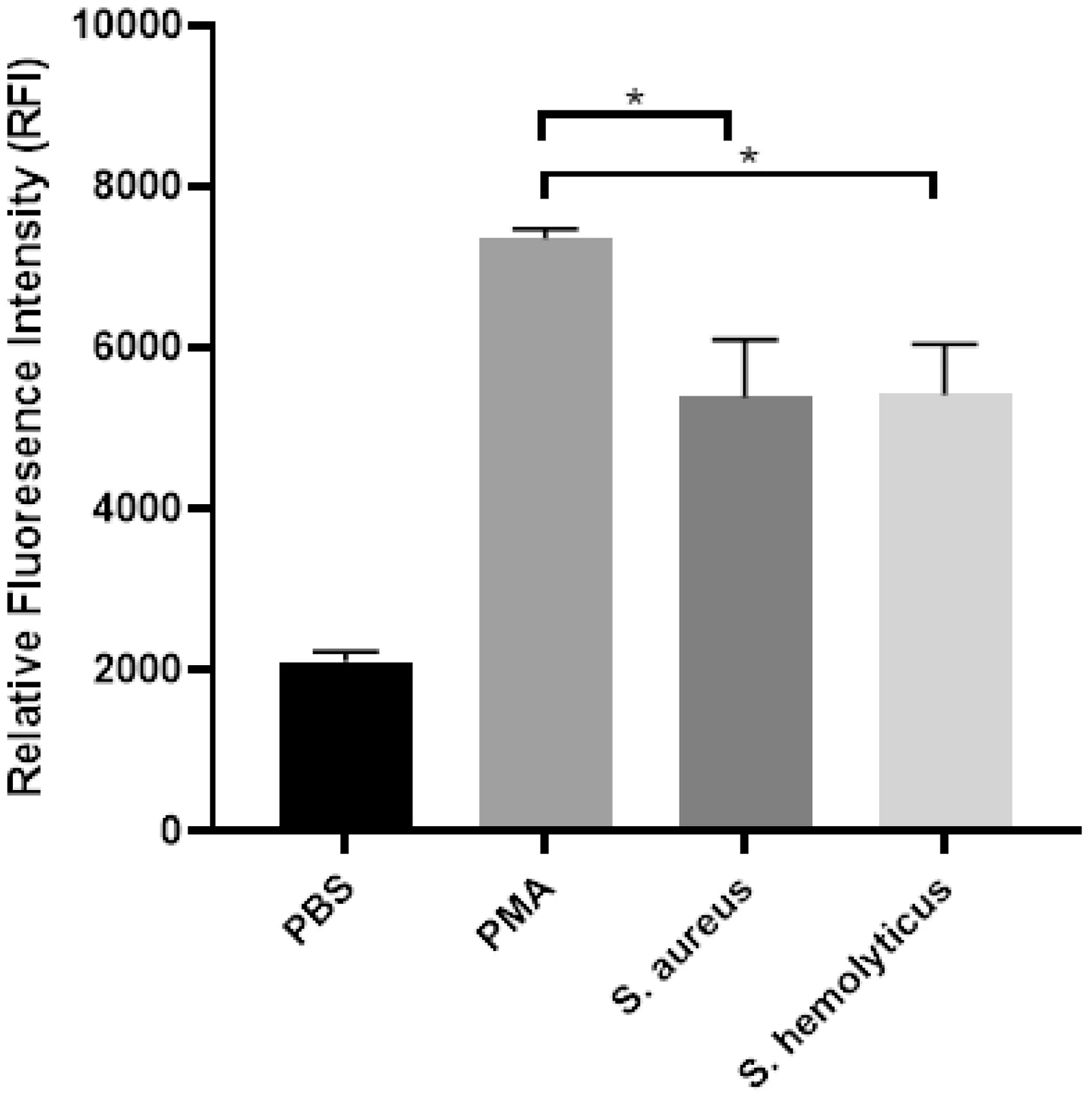
Quantification of NET-associated extracellular DNA release by SYTOX Green fluorometry. Neutrophils were left untreated or stimulated for 4 h with PMA, *S. aureus*, or *S. haemolyticus*. Extracellular DNA was stained with SYTOX Green, and fluorescence intensity was measured using a spectrofluorometer. Background fluorescence from bacteria-only wells was subtracted. Data are presented as mean ± standard error of the mean (SEM). *p* < 0.05 compared with PMA; Mann–Whitney U test.

### 3.6. *S. haemolyticus* resists NET-mediated killing

Because the preceding assays showed that *S. haemolyticus* can trigger NET-associated extracellular DNA release, we next examined whether this isolate was susceptible to NET-mediated antimicrobial activity. PMA-induced NETs were generated, and bacteria were added to wells containing intact NETs or MNase-treated NETs. MNase treatment was used to degrade NET-associated extracellular DNA, allowing estimation of the killing specifically attributable to NETs.

As shown in Figure 6a, fewer colonies were recovered from plates containing bacteria exposed to intact NETs compared with the corresponding MNase-treated controls. However, the extent of NET-mediated killing differed between the two bacterial species. NET-mediated killing of *S. haemolyticus* was markedly lower than that observed for the *S. aureus* comparator (15.64% vs. 45.70%, respectively; *p* = 0.002) (Figure 6b). These findings indicate that the tested clinical *S. haemolyticus* isolate was less susceptible to NET-mediated killing and suggest that it can resist NET-associated antimicrobial activity.

**Figure 6.**
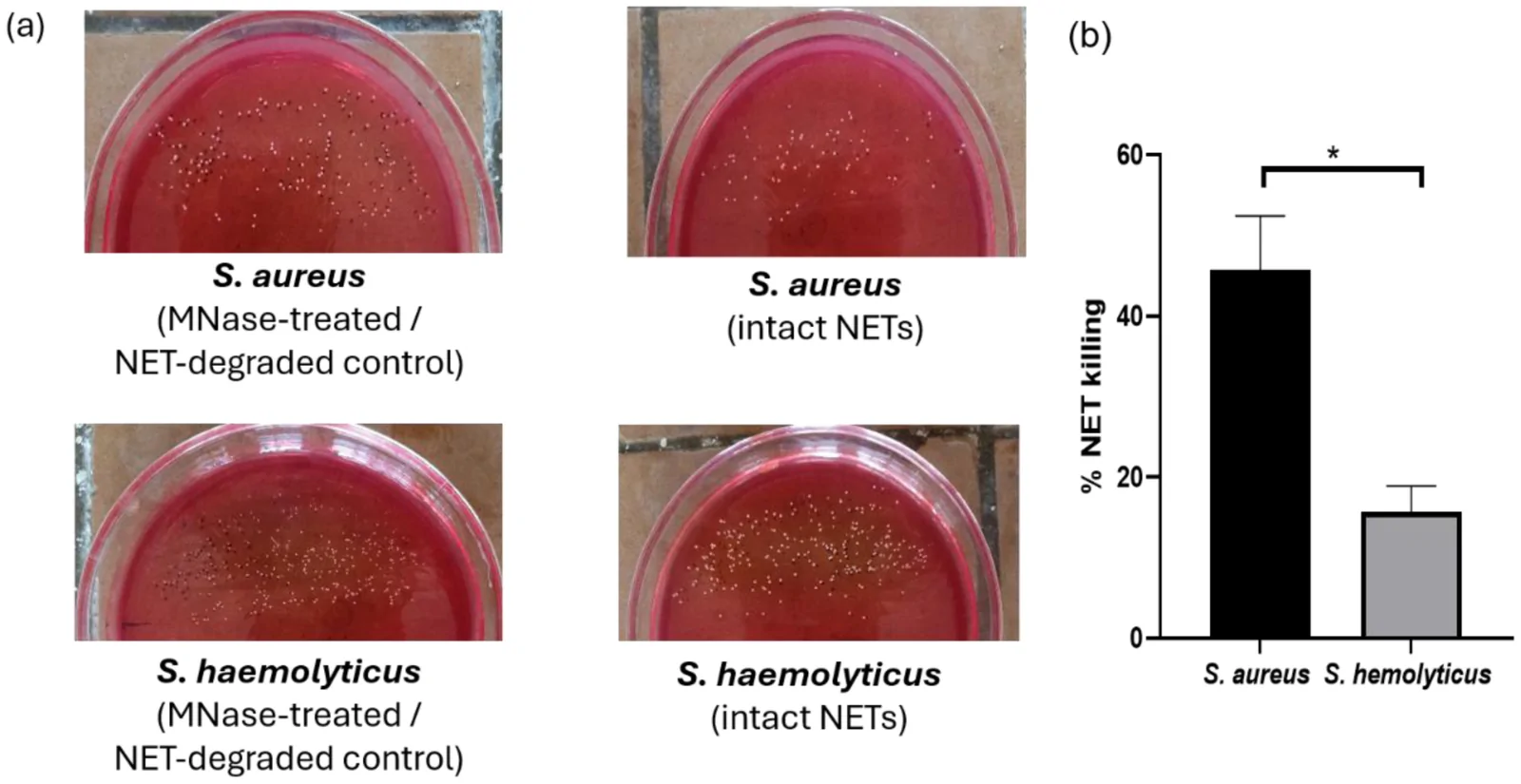
NET-mediated killing of *S. aureus* and *S. haemolyticus*. PMA-induced NETs were generated by stimulating neutrophils with 100 nM PMA for 3 h. Bacteria were then added and incubated for 1 h in wells containing intact NETs or MNase-treated NETs. (a) Representative agar plate images showing colony recovery after exposure to intact NETs or MNase-treated controls. (b) Quantification of NET-mediated killing percentage. Data are presented as mean ± SD. *p < 0.05, unpaired t-test.

### 3.7. NET-associated MPO-DNA complex formation and NE/MPO gene expression following bacterial stimulation

To further assess NET-associated DNA-protein complex formation, MPO-DNA complexes were quantified by capture ELISA. PBS-treated neutrophils showed minimal MPO-DNA complex formation, whereas PMA stimulation induced the strongest response, confirming robust NET-associated complex formation (Figure 7a). Bacterial exposure also increased MPO-DNA complex formation compared with PBS-treated neutrophils. MPO-DNA levels increased to 13.21 ± 1.56-fold following PMA stimulation, 7.15 ± 0.97-fold following exposure to *S. aureus*, and 5.20 ± 1.10-fold following exposure to *S. haemolyticus*. Although *S. haemolyticus* induced MPO-DNA complex formation, the magnitude of this response was lower than that observed with PMA and lower than that induced by the *S. aureus* comparator, supporting a comparatively attenuated NET-associated response.

Quantitative real-time PCR was then used to determine whether these NET-associated responses were accompanied by changes in the mRNA expression of neutrophil elastase (NE) and myeloperoxidase (MPO). As shown in Figure 7b,c, exposure to PMA, *S. aureus*, or *S. haemolyticus* did not significantly alter NE or MPO mRNA expression compared with PBS-treated neutrophils. These findings suggest that the observed differences in NET-associated responses were not associated with major transcriptional changes in NE or MPO under the experimental conditions used.

**Figure 7.**
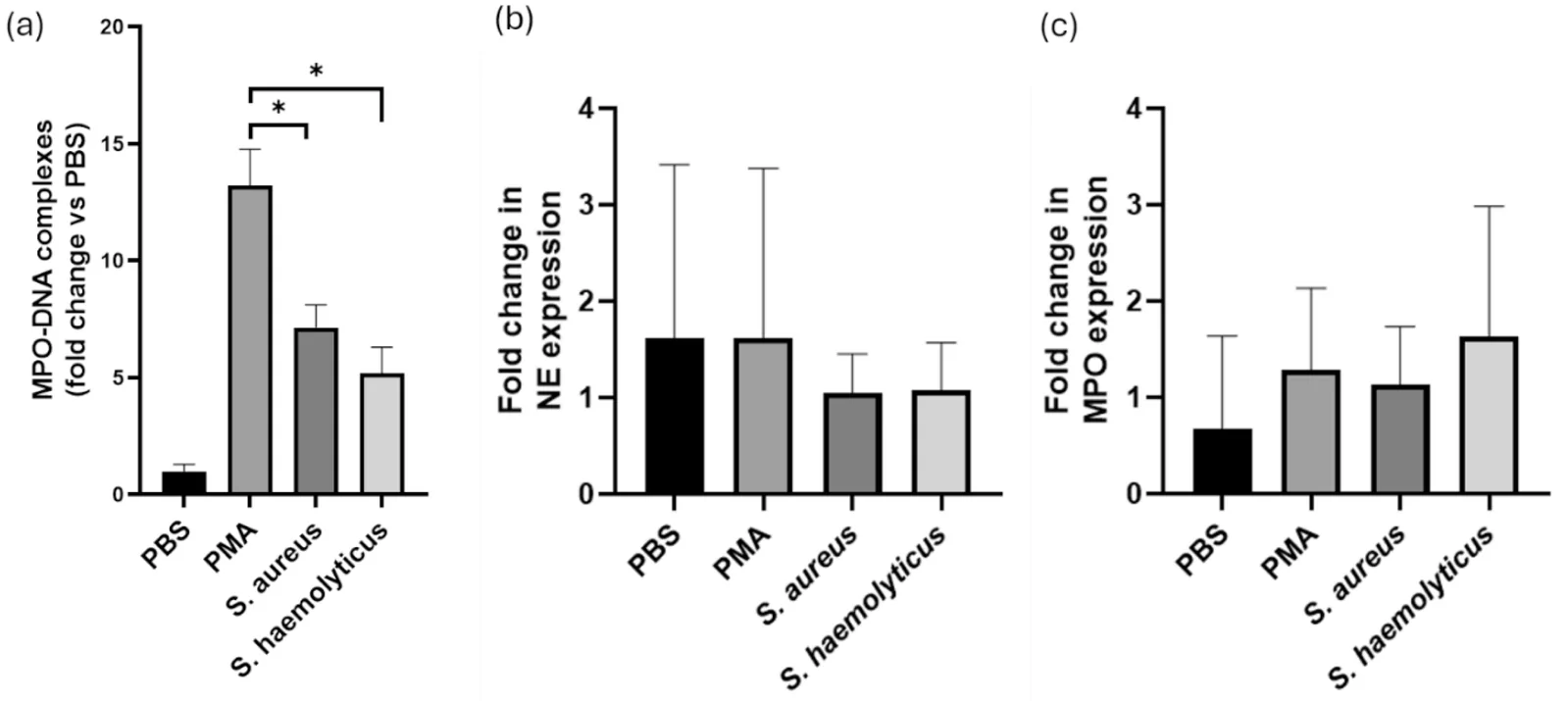
NET-associated MPO-DNA complex formation and NE/MPO mRNA expression following bacterial stimulation. Isolated human neutrophils were incubated for 4 h with PBS, PMA, *S. aureus*, or *S. haemolyticus*. (a) MPO-DNA complexes were quantified by capture ELISA and expressed as fold change relative to PBS-treated neutrophils. Data are presented as mean ± SD, n = 5. *p* < 0.05 compared with PMA-treated neutrophils, unpaired t-test. (b,c) Quantitative real-time PCR analysis of (b) neutrophil elastase (NE) and (c) myeloperoxidase (MPO) mRNA expression. Relative expression was normalized to GAPDH and expressed as fold change. Data are presented as mean ± SD.

## 4. Discussion

*S. haemolyticus* is increasingly recognized as an important opportunistic pathogen associated with healthcare-related infections. Despite its clinical relevance, the mechanisms that enable this species to survive within the host and persist in healthcare environments remain incompletely understood [33]. Hospital-associated *S. haemolyticus* strains are frequently characterized by extensive genomic plasticity, including a high abundance of insertion sequences, as well as multidrug resistance [34],[35]. These features may facilitate adaptation, persistence, and dissemination in clinical settings. Therefore, accurate identification and appropriate management of *S. haemolyticus* infections are important to limit the spread of resistant strains within healthcare facilities and, potentially, in the wider community [34].

Understanding how *S. haemolyticus* interacts with neutrophils is important for clarifying its pathogenic potential, particularly because its NET-associated responses remain poorly characterized. While *S. aureus* has been extensively studied for resistance to neutrophil antimicrobial mechanisms, much less is known about whether *S. haemolyticus* can withstand similar defenses. Therefore, we examined neutrophil killing, phagocytosis, NET-associated responses, and NET-mediated killing using a multidrug-resistant clinical *S. haemolyticus* isolate and a clinical *S. aureus* comparator.

The tested *S. haemolyticus* isolate showed higher survival after neutrophil exposure than the *S. aureus* comparator. Specifically, 64.0% of *S. haemolyticus* survived after incubation with neutrophils, compared with 27.6% of *S. aureus*. This finding suggests that the tested *S. haemolyticus* isolate was less susceptible to neutrophil-mediated killing. Several factors may contribute to this phenotype. *S. haemolyticus* has been reported to possess virulence-associated traits, including hemolytic activity, cytotoxin-associated genes, multidrug resistance, and biofilm-forming capacity, all of which may support persistence during infection [36]. Previous studies reported a high prevalence of hemolysin-associated genes among clinical *S. haemolyticus* isolates, suggesting that cytotoxic mechanisms may contribute to tissue damage and immune-cell interaction [37]. The presence of the *hla* gene is highly prevalent, being detected in over 90% of clinical *S. haemolyticus* strains [36],[37]. This toxin in *S. aureus* lyses and kills a variety of cell types because it binds to the host receptor ADAM-10 and then oligomerizes to create heptameric holes in host cell membranes, as mentioned by Tam K and Torres VJ [38]. Although the direct contribution of similar cytotoxic mechanisms to neutrophil resistance in the tested *S. haemolyticus* isolate was not examined here, such virulence-associated factors may partly explain its increased survival following neutrophil exposure, warranting further investigation.

Neutrophils are specialized phagocytes that detect, engulf, and destroy invading microorganisms. During phagocytosis, bacteria are internalized into membrane-bound phagosomes, which subsequently fuse with cytoplasmic granules to form phagolysosomes enriched with antimicrobial proteins, peptides, and reactive mediators [39]. In the present study, flow cytometric analysis showed that the tested *S. haemolyticus* isolate exhibited delayed early phagocytic uptake compared with the *S. aureus* comparator. At 10 min, phagocytosis of *S. haemolyticus* was significantly lower than that of *S. aureus* (13.3% vs. 29.2%, respectively). At 30 min, uptake of *S. haemolyticus* remained lower, although the difference was not statistically significant. By 60 min, phagocytosis became comparable between the two species.

This delayed early uptake may contribute to the increased survival of *S. haemolyticus* following neutrophil exposure. However, because phagocytosis became comparable at the later time point, reduced uptake alone is unlikely to fully explain the higher survival of *S. haemolyticus*. This suggests that additional mechanisms, such as resistance to intracellular killing or tolerance to extracellular antimicrobial mechanisms, may also contribute to its persistence under neutrophil-mediated immune pressure.

Several staphylococcal virulence factors are known to interfere with neutrophil antimicrobial activity. In *S. aureus*, surface-associated proteins such as fibronectin-binding proteins, clumping factor A, and other adhesins can influence host–cell interaction and may reduce phagocytic clearance [40]. In addition, *S. aureus* possesses multiple defenses against reactive oxygen and nitrogen species, including flavohemoglobin, catalase, superoxide dismutases, staphyloxanthin, and alkyl hydroperoxide reductase, some of which are upregulated following neutrophil phagocytosis [41]. Although these mechanisms are best characterized in *S. aureus*, analogous or distinct immune-evasion strategies may contribute to the delayed early uptake and increased neutrophil survival of the tested clinical *S. haemolyticus* isolate.

Similar to *S. aureus*, *S. haemolyticus* can express surface-associated factors that promote bacterial adhesion and biofilm formation, which may indirectly influence its interaction with neutrophils. Previous studies have reported that *S. haemolyticus* biofilm development can involve extracellular DNA, proteins, and surface-associated adhesins, including factors related to elastin- and fibronectin-binding activity [42],[43]. In addition, emerging virulence-associated genes such as *sasX*, *sesI*, and *shsA* have been detected in nosocomial *S. haemolyticus* isolates. Silva-de-Jesus et al. suggested that the presence of *sasX/sesI/shsA*-like genes in *S. haemolyticus* may reflect horizontal gene transfer among staphylococcal species, including *S. aureus*, *S. epidermidis*, and *S. haemolyticus* [44]. Collectively, these virulence factors may contribute to the reduced early phagocytic uptake observed for *S. haemolyticus*.

Additionally, neutrophils can create extracellular traps to capture extracellular microorganisms [45]. Neutrophil extracellular traps (NETs) resemble webs composed of histones, chromatin, and neutrophil granule proteins that are released in response to specific stimuli or conditions [39]. Decondensed chromatin functions as a net that traps and eliminates microbes; however, the direct microbicidal activity of NETs against captured pathogens remains controversial [46]. This is particularly relevant for staphylococci, which have evolved multiple strategies to tolerate or evade NET-associated antimicrobial activity.

The present study provides new insight into the interaction between *S. haemolyticus* and human neutrophils, particularly in relation to NET-associated extracellular DNA release and resistance to NET-mediated killing. Although *S. haemolyticus* is often considered part of the normal skin microbiota, its association with healthcare-related infections and multidrug resistance highlights its pathogenic potential.

NET-associated responses were assessed using SEM, SYTOX Green fluorescence microscopy, microscopic quantification, fluorometric extracellular DNA measurement, and MPO-DNA complex ELISA. Both *S. aureus* and *S. haemolyticus* induced extracellular DNA release compared with PBS-treated neutrophils, indicating activation of NET-associated responses. However, PMA induced the strongest response, while *S. haemolyticus* induced a more moderate response than the *S. aureus* comparator. Previous studies have shown that *S. aureus* can induce NET formation through intact bacterial cells or secreted factors such as Panton-Valentine leucocidin [47],[48]. Although the mechanisms involved in *S. haemolyticus*–induced NET-associated responses remain undefined, our findings suggest that this species can engage neutrophil extracellular DNA-release pathways. Further studies are needed to identify the bacterial and host factors responsible for this response.

Several *S. aureus* molecules have been implicated in NET induction. For example, leukotoxin LukGH/LukAB can stimulate NET release and promote trapping of *S. aureus* cells without necessarily leading to efficient bacterial killing [49]. Björnsdottir, Halla, et al. reported that phenol-soluble modulin a (PSMa) stimulates rapid NET development via a ROS-independent mechanism [50], whereas Hoppenbrouwers, Tamara, et al. reported staphylococcal protein A as an important contributor to NET formation [51]. These studies indicate that staphylococci can actively modulate NET formation through different secreted or surface-associated factors.

NETs can limit microbial dissemination by physically trapping pathogens and concentrating antimicrobial molecules at the site of infection [45],[52],[53]. In 2004, Arturo Zychlinsky and his team described these structures as important functional components of the immune system [45]. The mechanisms through which NETs combat and ultimately eliminate microbial invaders are still debatable. Menegazzi et al. reported that NETs trap *S. aureus* and *Candida albicans* but do not kill them when they are captured by these structures [46]. This concept is consistent with our findings, in which the tested *S. haemolyticus* isolate induced NET-associated responses but showed relatively high resistance to NET-mediated killing.

In contrast, other studies support a direct antimicrobial role for NETs. NET-associated DNA may contribute to bacterial killing by binding surface-associated cations, disrupting membrane integrity, and promoting bacterial lysis [54]. In addition, antimicrobial peptides such as LL-37 can protect NET-derived DNA from bacterial nuclease-mediated degradation [55]. NET-associated proteins, particularly histones, have also been implicated in the antimicrobial function of NETs [56],[57] and the zinc-chelating protein calprotectin [58]. In addition, MPO bound to NETs exerts antimicrobial effects necessary for eliminating pathogens when hydrogen peroxide is present [59]. In the present study, NET-mediated killing of the tested *S. haemolyticus* isolate was markedly lower than that of the *S. aureus* comparator, suggesting that *S. haemolyticus* can withstand NET-associated antimicrobial components despite inducing NET-associated extracellular DNA release.

In the present study, the tested *S. haemolyticus* isolate showed markedly lower susceptibility to NET-mediated killing than the *S. aureus* comparator, with NET-mediated killing percentages of 15.64% and 45.70%, respectively. This finding suggests that *S. haemolyticus* can tolerate NET-associated antimicrobial activity more effectively than *S. aureus* under the experimental conditions used. Together with the moderate NET-associated extracellular DNA release observed after bacterial stimulation, these results support the possibility that *S. haemolyticus* possesses immune-evasion strategies that allow persistence under neutrophil-mediated immune pressure. This is consistent with previous reports showing that coagulase-negative staphylococci can withstand neutrophil antimicrobial mechanisms through diverse survival strategies [60],[61].

*S. haemolyticus* may express extracellular enzymes such as nucleases or proteases that degrade NET structures or neutralize their antimicrobial components like *S. aureus*. For example, Berends et al. demonstrated that *S. aureus* uses its nuclease (*nuc*) to evade entrapment by NETs [62]. In addition, Thammavongsa et al. showed that when *S. aureus* nuclease and adenosine synthase A are combined, NETs are transformed into deoxyadenosine, which causes immune cells to die [63].

Eisenbeis, Janina, et al. reported that *S. aureus* blocks NETs formation and their activities by extracellular adherence protein (Eap). Through atomic force microscopy, it was demonstrated that Eap binds and aggregates linearized DNA. In line with this, Eap prevents the formation of NETs, suggesting a potential role in protecting bacteria from capture by structures like microthrombi [64]. Stapels, Daphne AC, et al. reported that Eap and its homologs Ehp1 and Ehp2 strongly inhibit the neutrophil elastase (NE), neutrophil serine proteases (NSPs), cathepsin G, and proteinase 3 [65].

Biofilm-associated factors may also contribute to protection against NET-mediated antimicrobial activity. Biofilm matrix components can limit the penetration of immune effectors and enhance bacterial persistence [66],[67]. This is particularly relevant for *S. haemolyticus*, which is well recognized for its ability to form biofilms on medical devices and in hospital-associated infections. Previous studies have shown that *S. haemolyticus* biofilms often contain abundant extracellular DNA and proteins, in contrast to biofilms of some other staphylococcal species that are more dependent on polysaccharide intercellular adhesin [68]. In addition, some strains produce more capsular polysaccharides, which results in a mucoid phenotype linked to higher virulence [69].

A related mechanism has been described for fibronectin-binding protein B (FnBPB), which can protect staphylococci from histone-mediated antimicrobial activity. Pietrocola et al. reported that FnBPB binds histone H3 with high affinity. An *fnbB* knockout mutant showed reduced H3 binding and increased susceptibility to histone-mediated killing, whereas FnBPB overexpression enhanced H3 binding and increased resistance to the bactericidal effects of histones. Importantly, FnBPB also protected bacteria in a NET-mediated killing assay, highlighting the relevance of histone resistance under NET-associated antimicrobial conditions [70].

To explore whether the observed NET-associated phenotype was accompanied by transcriptional changes in key NET-associated enzymes, we measured NE and MPO mRNA expression by quantitative real-time PCR. PMA, *S. aureus*, and *S. haemolyticus* did not significantly alter NE or MPO mRNA expression after 4 h of stimulation. This suggests that the differences observed in extracellular DNA release, MPO-DNA complex formation, and NET-mediated killing were not explained by increased transcription of these enzymes under the experimental conditions used.

This finding is consistent with previous reports showing that PMA does not markedly increase NE or MPO mRNA expression, although it may affect other NET-related genes such as *PAD4* [32]. It also agrees with the concept that mature neutrophils contain preformed granule proteins required for NET formation and that NET release can occur independently of de novo gene transcription or protein synthesis [71]. Therefore, the reduced susceptibility of the tested *S. haemolyticus* isolate to NET-mediated killing is more likely related to bacterial resistance mechanisms or post-transcriptional neutrophil responses than altered NE or MPO gene expression.

Although the present study combined SEM, extracellular DNA quantification, microscopic enumeration, and MPO-DNA complex ELISA to assess NET-associated responses, direct immunofluorescence co-localization of extracellular DNA with NET-associated proteins such as MPO, neutrophil elastase, or citrullinated histone H3 was not performed. Future studies using direct co-localization approaches, larger collections of clinical isolates, and bacterial mutant or gene-expression analyses will help define the molecular composition of these structures and determine whether the observed phenotype is conserved among *S. haemolyticus* strains.

## 5. Conclusions

The tested multidrug-resistant clinical *Staphylococcus haemolyticus* isolate showed reduced susceptibility to neutrophil-mediated killing and NET-mediated antimicrobial activity compared with the clinical *Staphylococcus aureus*. Although *S. haemolyticus* induced extracellular DNA release and MPO-DNA complex formation, these responses were lower than those induced by PMA, indicating an attenuated NET-associated response. NE and MPO mRNA expression was not significantly altered, suggesting that transcriptional regulation of these enzymes is unlikely to explain the observed phenotype. Overall, these findings provide initial evidence that *S. haemolyticus* can modulate NET-associated neutrophil responses while resisting NET-mediated killing. Further studies using larger collections of clinical isolates are needed to determine whether this phenotype is conserved among *S. haemolyticus* strains and to identify the underlying mechanisms.

## Use of AI tools declaration

The authors declare they have not used Artificial Intelligence (AI) tools in the creation of this article.

## References

[1] Becker K, Heilmann C, Peters G (2014). Coagulase-negative staphylococci. Clin Microbiol Rev.

[2] Becker K, Both A, Weißelberg S (2020). Emergence of coagulase-negative staphylococci. Expert Rev Anti Infect Ther.

[3] Czekaj T, Ciszewski M, Szewczyk EM (2015). *Staphylococcus haemolyticus*—an emerging threat in the twilight of the antibiotics age. Microbiology.

[4] Djawadi B, Heidari N, Mohseni M (2023). UTI caused by *Staphylococcus saprophyticus*. Urinary Tract Infections—New Insights.

[5] Severn MM, Horswill AR (2023). *Staphylococcus epidermidis* and its dual lifestyle in skin health and infection. Nat Rev Microbiol.

[6] Eltwisy HO, Twisy HO, Hafez MHR (2022). Clinical infections, antibiotic resistance, and pathogenesis of *Staphylococcus haemolyticus*. Microorganisms.

[7] Ahmed A, Satti L, Zaman G (2019). Catheter related recurrent blood stream infection caused by linezolid-resistant, methicillin resistant *Staphylococcus haemolyticus*; an emerging super bug. J Pak Med Assoc.

[8] Chang PH, Liu TP, Huang PY (2018). Clinical features, outcomes, and molecular characteristics of an outbreak of *Staphylococcus haemolyticus* infection, among a mass-burn casualty patient group, in a tertiary center in northern Taiwan. J Microbiol Immunol Infect.

[9] Matsuzono K, Ishiyama Y, Higaki A (2021). Successful endovascular coiling of infectious cerebral aneurysm following *Staphylococcus haemolyticus* endocarditis. J Int Med Res.

[10] Westberg R, Stegger M, Söderquist B (2022). Molecular epidemiology of neonatal-associated *Staphylococcus haemolyticus* reveals endemic outbreak. Microbiol Spectr.

[11] Natsis NE, Cohen PR (2018). Coagulase-negative Staphylococcus skin and soft tissue infections. Am J Clin Dermatol.

[12] Puca V, Marulli RZ, Grande R (2021). Microbial species isolated from infected wounds and antimicrobial resistance analysis: Data emerging from a three-years retrospective study. Antibiotics.

[13] Haque MH, Miah ML, Sarker S (2021). Molecular characterization and antibiogram profiling of multidrug resistant *Staphylococcus haemolyticus* isolated from patients with urinary tract infection in Bangladesh. J Bacteriol Mycol.

[14] Phillip S, Mushi MF, Decano AG (2023). Molecular characterizations of the coagulase-negative *staphylococci* species causing urinary tract infection in Tanzania: A laboratory-based cross-sectional study. Pathogens.

[15] Mizuno S, Fujikawa T, Uemura S (2023). Nosocomial meningitis caused by *Staphylococcus haemolyticus* in a child with neutropenia in the absence of intracranial devices: A case report. BMC Infect Dis.

[16] Panda S, Kar S, Sharma S (2016). Multidrug-resistant *Staphylococcus haemolyticus* isolates from infected eyes and healthy conjunctivae in India. J Glob Antimicrob Resist.

[17] Michalik M, Kosecka-Strojek M, Wolska M (2021). First case of *staphylococci* carrying linezolid resistance genes from laryngological infections in Poland. Pathogens.

[18] DeLeo FR, Nauseef WM (2014). Granulocytic phagocytes. Mandell, Douglas, and Bennet's Principles and Practice of Infectious Diseases.

[19] Nathan C (2006). Neutrophils and immunity: Challenges and opportunities. Nat Rev Immunol.

[20] Papayannopoulos V (2018). Neutrophil extracellular traps in immunity and disease. Nat Rev Immunol.

[21] Wartha F, Beiter K, Normark S (2007). Neutrophil extracellular traps: Casting the NET over pathogenesis. Curr Opin Microbiol.

[22] Medina E (2009). Neutrophil extracellular traps: A strategic tactic to defeat pathogens with potential consequences for the host. J Innate Immun.

[23] Muñiz-Buenrostro A, Arce-Mendoza AY, Montes-Zapata EI (2020). Simplified neutrophil isolation protocol. Int J Immunol Immunother.

[24] Jin L, Liu Y, Jing C (2020). Neutrophil extracellular traps (NETs)-mediated killing of carbapenem-resistant hypervirulent *Klebsiella pneumoniae* (CR-hvKP) are impaired in patients with diabetes mellitus. Virulence.

[25] Heinzelmann M, Gardner SA, Mercer-Jones M (1999). Quantification of phagocytosis in human neutrophils by flow cytometry. Microbiol Immunol.

[26] Wang L, Shen D, Wu H (2017). Resistance of hypervirulent *Klebsiella pneumoniae* to both intracellular and extracellular killing of neutrophils. PLoS One.

[27] White PC, Chicca IJ, Ling MR (2017). Characterization, quantification, and visualization of neutrophil extracellular traps. Oral Biology: Molecular Techniques and Applications.

[28] Jiang D, Saffarzadeh M, Scharffetter-Kochanek K (2017). *In vitro* demonstration and quantification of neutrophil extracellular trap formation. Bio-protocol.

[29] Bystrzycka W, Manda-Handzlik A, Sieczkowska S (2017). Azithromycin and chloramphenicol diminish neutrophil extracellular traps (NETs) release. Int J Mol Sci.

[30] Yoo DG, Floyd M, Winn M (2014). NET formation induced by *Pseudomonas aeruginosa* cystic fibrosis isolates measured as release of myeloperoxidase-DNA and neutrophil elastase-DNA complexes. Immunol Lett.

[31] Floyd M, Winn M, Cullen C (2016). Swimming motility mediates the formation of neutrophil extracellular traps induced by flagellated *Pseudomonas aeruginosa*. PLoS Pathog.

[32] Agraz-Cibrian JM, Giraldo DM, Urcuqui-Inchima S (2019). 1,25-Dihydroxyvitamin D3 induces formation of neutrophil extracellular trap-like structures and modulates the transcription of genes whose products are neutrophil extracellular trap-associated proteins: A pilot study. Steroids.

[33] Cavanagh JP, Hjerde E, Holden MTG (2014). Whole-genome sequencing reveals clonal expansion of multiresistant *Staphylococcus haemolyticus* in European hospitals. J Antimicrob Chemother.

[34] Hosseinkhani F, Jabalameli F, Farahani NN (2016). Variable number of tandem repeat profiles and antimicrobial resistance patterns of *Staphylococcus haemolyticus* strains isolated from blood cultures in children. Infect Genet Evol.

[35] Ahmed NH, Baruah FK, Grover RK (2015). Staphylococcal blood stream infections in cancer patients. Ann Med Health Sci Res.

[36] Pinheiro L, Ivo Brito C, De Oliveira A (2015). *Staphylococcus epidermidis* and *Staphylococcus haemolyticus*: Molecular detection of cytotoxin and enterotoxin genes. Toxins.

[37] Nasaj M, Saeidi Z, Asghari B (2020). Identification of hemolysin encoding genes and their association with antimicrobial resistance pattern among clinical isolates of coagulase-negative *Staphylococci*. BMC Res Notes.

[38] Tam K, Torres VJ (2019). *Staphylococcus aureus* secreted toxins and extracellular enzymes. Microbiol Spectr.

[39] Rungelrath V, DeLeo FR (2021). *Staphylococcus aureus*, antibiotic resistance, and the interaction with human neutrophils. Antioxid Redox Signal.

[40] Higgins J, Loughman A, Van Kessel KPM (2006). Clumping factor A of *Staphylococcus aureus* inhibits phagocytosis by human polymorphonuclear leucocytes. FEMS Microbiol Lett.

[41] Voyich JM, Braughton KR, Sturdevant DE (2005). Insights into mechanisms used by *Staphylococcus aureus* to avoid destruction by human neutrophils. J Immunol.

[42] Downer R, Roche F, Park PW (2002). The elastin-binding protein of *Staphylococcus aureus* (EbpS) is expressed at the cell surface as an integral membrane protein and not as a cell wall-associated protein. J Biol Chem.

[43] Panda S, Singh DV (2018). Biofilm formation by ica-negative ocular isolates of *Staphylococcus haemolyticus*. Front Microbiol.

[44] C Silva-de-Jesus A, Rossi CC, Pereira-Ribeiro PMA (2023). Unusual carriage of virulence genes sasX/sesI/shsA by nosocomial *Staphylococcus haemolyticus* from Brazil. Future Microbiol.

[45] Brinkmann V, Reichard U, Goosmann C (2004). Neutrophil extracellular traps kill bacteria. Science.

[46] Menegazzi R, Decleva E, Dri P (2012). Killing by neutrophil extracellular traps: Fact or folklore?. Blood.

[47] Pilsczek FH, Salina D, Poon KK (2010). A novel mechanism of rapid nuclear neutrophil extracellular trap formation in response to *Staphylococcus aureus*. J Immunol.

[48] Mazzoleni V, Zimmermann K, Smirnova A (2021). *Staphylococcus aureus* Panton-Valentine Leukocidin triggers an alternative NETosis process targeting mitochondria. FASEB J.

[49] Malachowa N, Kobayashi SD, Freedman B (2013). *Staphylococcus aureus* leukotoxin GH promotes formation of neutrophil extracellular traps. J Immunol.

[50] Björnsdottir H, Dahlstrand Rudin A, Klose FP (2017). Phenol-soluble modulin α peptide toxins from aggressive *Staphylococcus aureus* induce rapid formation of neutrophil extracellular traps through a reactive oxygen species-independent pathway. Front Immunol.

[51] Hoppenbrouwers T, Sultan AR, Abraham TE (2018). *Staphylococcal* protein A is a key factor in neutrophil extracellular traps formation. Front Immunol.

[52] Brinkmann V, Zychlinsky A (2007). Beneficial suicide: Why neutrophils die to make NETs. Nat Rev Microbiol.

[53] Papayannopoulos V, Zychlinsky A (2009). NETs: A new strategy for using old weapons. Trends Immunol.

[54] Halverson TWR, Wilton M, Poon KKH (2015). DNA is an antimicrobial component of neutrophil extracellular traps. PLoS Pathog.

[55] Neumann A, Völlger L, Berends E (2014). Novel role of the antimicrobial peptide LL-37 in the protection of neutrophil extracellular traps against degradation by bacterial nucleases. J Innate Immun.

[56] Richards RC, O'Neil DB, Thibault P (2001). Histone H1: An antimicrobial protein of Atlantic salmon (Salmo salar). Biochem Biophys Res Commun.

[57] Parseghian MH, Luhrs KA (2006). Beyond the walls of the nucleus: The role of histones in cellular signaling and innate immunity. Biochem Cell Biol.

[58] Urban CF, Ermert D, Schmid M (2009). Neutrophil extracellular traps contain calprotectin, a cytosolic protein complex involved in host defense against *Candida albicans*. PLoS Pathog.

[59] Parker H, Albrett AM, Kettle AJ (2012). Myeloperoxidase associated with neutrophil extracellular traps is active and mediates bacterial killing in the presence of hydrogen peroxide. J Leukoc Biol.

[60] Otto M (2009). *Staphylococcus epidermidis*—the ‘accidental’ pathogen. Nat Rev Microbiol.

[61] Spiliopoulou AI, Krevvata MI, Kolonitsiou F (2012). An extracellular *Staphylococcus epidermidis* polysaccharide: Relation to polysaccharide intercellular adhesin and its implication in phagocytosis. BMC Microbiol.

[62] Berends ETM, Horswill AR, Haste NM (2010). Nuclease expression by *Staphylococcus aureus* facilitates escape from neutrophil extracellular traps. J Innate Immun.

[63] Thammavongsa V, Missiakas DM, Schneewind O (2013). *Staphylococcus aureus* degrades neutrophil extracellular traps to promote immune cell death. Science.

[64] Eisenbeis J, Saffarzadeh M, Peisker H (2018). The *Staphylococcus aureus* extracellular adherence protein Eap is a DNA binding protein capable of blocking neutrophil extracellular trap formation. Front Cell Infect Microbiol.

[65] Stapels DAC, Ramyar KX, Bischoff M (2014). *Staphylococcus aureus* secretes a unique class of neutrophil serine protease inhibitors. Proc Natl Acad Sci USA.

[66] Christner M, Franke GC, Schommer NN (2010). The giant extracellular matrix-binding protein of *Staphylococcus epidermidis* mediates biofilm accumulation and attachment to fibronectin. Mol Microbiol.

[67] Cerca N, Jefferson KK, Oliveira R (2006). Comparative antibody-mediated phagocytosis of *Staphylococcus epidermidis* cells grown in a biofilm or in the planktonic state. Infect Immun.

[68] Fredheim EGA, Klingenberg C, Rohde H (2009). Biofilm formation by *Staphylococcus haemolyticus*. J Clin Microbiol.

[69] Singhal L, Gupta V, Sharma S (2023). Mucoid *Staphylococcus haemolyticus*: An unheeded multidrug-resistant pathogen. Braz J Microbiol.

[70] Pietrocola G, Nobile G, Alfeo MJ (2019). Fibronectin-binding protein B (FnBPB) from *Staphylococcus aureus* protects against the antimicrobial activity of histones. J Biol Chem.

[71] Sollberger G, Amulic B, Zychlinsky A (2016). Neutrophil extracellular trap formation is independent of *de novo* gene expression. PLoS One.

